# MRI Superresolution Using Self-Similarity and Image Priors

**DOI:** 10.1155/2010/425891

**Published:** 2010-12-08

**Authors:** José V. Manjón, Pierrick Coupé, Antonio Buades, D. Louis Collins, Montserrat Robles

**Affiliations:** ^1^Instituto de Aplicaciones de las Tecnologías de la Información y de las Comunicaciones Avanzadas (ITACA), Universidad Politécnica de Valencia, Camino de Vera s/n, 46022 Valencia, Spain; ^2^McConnell Brain Imaging Centre, Montreal Neurological Institute, McGill University, Montreal, Canada H3A 2B4; ^3^Mathématiques et Informatique, Université Paris Descartes, 45 Rue des Saints Pères, 75270 Paris Cedex 06, France; ^4^Department de Matemàtiques i Informàtica, Universitat Illes Balears, Ctra Valldemossa km 7.5, 07122 Palma de Mallorca, Spain

## Abstract

In Magnetic Resonance Imaging typical clinical settings, both low- and high-resolution images of different types are routinarily acquired. In some cases, the acquired low-resolution images have to be upsampled to match with other high-resolution images for posterior analysis or postprocessing such as registration or multimodal segmentation. However, classical interpolation techniques are not able to recover the high-frequency information lost during the acquisition process. In the present paper, a new superresolution method is proposed to reconstruct high-resolution images from the low-resolution ones using information from coplanar high resolution images acquired of the same subject. Furthermore, the reconstruction process is constrained to be physically plausible with the MR acquisition model that allows a meaningful interpretation of the results. Experiments on synthetic and real data are supplied to show the effectiveness of the proposed approach. A comparison with classical state-of-the-art interpolation techniques is presented to demonstrate the improved performance of the proposed methodology.

## 1. Introduction

In Magnetic Resonance Imaging (MRI), data is acquired with a finite resolution that is limited by several factors such as the Signal-to-Noise Ratio (SNR), hardware and time limitations or patient's comfort. In typical clinical settings, several types of images are obtained with different voxel resolutions. Traditionally, in-plane resolution has been higher than resolution in the slice direction yielding nonisotropic voxel sizes.

In many applications, such as image segmentation or registration, data has to be upsampled to decrease its voxel size to make it compatible with a higher-resolution dataset [1, 2]. In such cases, interpolation techniques [3, 4] have been traditionally applied. Techniques such as linear interpolation or spline-based methods have been used extensively in the past to decrease voxel size and increase apparent data resolution. However, such techniques estimate new points assuming that the existing ones (in the low-resolution (LR) image) have the same value in the high-resolution (HR) images which is only valid within homogeneous regions. As a result, interpolated images are typically blurred versions of the underlying HR images.

A better approach to increase effectively the resolution of the reconstructed data is to use Superresolution (SR) techniques [5]. In MRI, superresolution techniques have been previously applied to increase image resolution in functional MRI (fMRI) [6] and Diffusion Tensor Imaging (DTI) studies [7]. Unfortunately, most of such techniques are based on the acquisition of multiple low-resolution images with small shifts, a process which is time consuming and therefore not adequate for typical clinical settings. 

Fortunately, if HR images of the same subject within the same or other image modality are available, it is possible to recover some of the lost high-frequency information within the LR image. This idea was recently applied in a method proposed by Rousseau [8] where a low-resolution volume is reconstructed using information of an HR reference volume while taking into account an expected degradation model. In Rousseau's method the HR data is used to regularize a deconvolution-based reconstruction using a Nonlocal Means denoising method [9]. The method proposed in this paper is related to Rousseau's work in the sense that we also use HR data to constrain the reconstruction process but our method is based on a totally different strategy to compute the image reconstruction.

## 2. Material and Methods

In MRI, image voxels in LR data *y* can be related to the corresponding underlying HR voxels *x* through a simple degradation model:

(1) y=DHx+n,

where *D* is a decimation operator (defined as taking each *L*th value starting from zero in each dimension), *H* is the convolution matrix, *x* is the underlying HR data, and *n* is a Rician distributed random noise [10]. In MRI, *H* can be roughly approximated by a 3D boxcar function since the values on LR data can be well modeled as an average of the corresponding HR voxel values. Therefore, the value *y*_*j*_ of any voxel in the LR image can be expressed as follows:

(2) $$\;y_{j}=\frac{1}{N}\overset{N}{\underset{i=1}{\sum }}x_{i}+n,$$

where the value of the LR voxel *y*_*j*_ is the average of the corresponding *Nx*_*i*_ voxels in the subjacent HR image plus some noise from the measurement process. 

Therefore, the aim of a superresolution method is to find the *x*_*i*_ values from the *y*_*j*_ values. This is a very ill-posed problem since there are infinite *x*_*i*_ values that meet such a condition. A common approach to solve this problem is to minimize a merit function such as

(3) $$\hat{x}=\underset{x}{\arg\;\min\;}\;{||y-DHx||}^{2}.$$

Due to the nonuniqueness of the solution for this problem, extra information is needed to constrain the possible solutions of (3) to obtain plausible results. One commonly used approach is to apply smoothness constrains in the reconstruction process that are based on the assumption of smoothness of the reconstructed data:

(4) $$\hat{x}=\underset{x}{\arg\;\min\;}\;\left({||y-DHx||}^{2}+\lambda R\left(x\right)\right),$$

where *R*(*x*) is a regularization term and *λ* is a weight that balances the contribution of smoothness and data fidelity terms. However, such smoothness assumption penalizes high-frequency content of the reconstructed image that is precisely what we want to obtain. 

In contrast to this optimization approaches, we propose to estímate $\hat{x}$ using a direct iterative method using coplanar HR data to control the reconstruction process. We prefer an iterative reconstruction-correction scheme to avoid classical optimization problems such as local minima and parameter initializations.

### 2.1. Proposed Method

The method proposed in this paper is not based on the smoothness assumption but on the assumption that if a registered HR image/volume of the same subject from the same or other modality is available, anatomical information from this HR data can be used to recover some image details in the superresolution-reconstructed LR data. Furthermore, by applying a specific filter to minimize the noise present in the LR data, we can impose as an additional constraint the fact that the downsampled version of the reconstructed data has to be exactly the same as the original LR data. This constraint has been previously applied in the SR context and referred as *subsampling consistency* [11]:

(5) $$y-DH\hat{x}=0.$$

To apply the proposed method, two preprocessing steps have to be performed.

- *Data Registration.* In order to extrapolate voxel local similarities from the HR reference data to the LR-reconstructed data both HR reference and LR data must be in the same geometrical space.
- *Image Denoising.* Due to the presence of noise, the constrain expressed in (5) cannot be directly used. To simplify the problem, LR data is first denoised using a recently proposed robust denoising method for 3D MR images [12] based in the well-known Nonlocal Means filter early proposed by Buades et al. [9]. To deal with bias introduced by the Rician nature of the noise in MR, the Rician adaptation proposed in [13] has been used. It has been demonstrated that such filter (i.e., the BNLM3D filter) is able to remove noise effectively while minimally affecting the image structure. Additionally the HR data is also filtered using the same filter to allow computing voxel similarities in a voxelwise manner which reduces the computational burden of the method while it increases the number of similar voxels (the comparison of voxel intensities is rotationally invariant while this is not the case of patch-based comparison).

The proposed method uses as input data an HR reference data and a preinterpolated version of the LR data. It is an iterative procedure based on two steps that corresponds to the two assumptions used which are the following.

(1)* Reconstruction.* Locally, similar voxels in the HR data tend to be similar in the reconstructed LR data. Therefore, averaging voxels in the interpolated version of the LR data using as reference the HR data similarities will enforce this condition. This is the key contribution of the proposed procedure. First, the LR data is interpolated in order to obtain a volume with the same voxel size as the HR reference data. To reconstruct the interpolated data, a 3D Neighborhood filter [14] could be used. However, the weights would not be calculated using the reference HR data information jointly with the LR data. The inclusion of LR data information in the reconstruction process allows the method to be robust to small misalignments between LR and HR data and also allows a coherent reconstruction even in cases when a feature is not visible in HR data by using LR self-similarity to help in the reconstruction process:

(6) $${\hat{x}}_{p}^{t+1}=\frac{1}{C_{p}}\overset{}{\underset{\forall q\in \Omega }{\sum }}{\hat{x}}_{q}^{t}e^{-{\left(z_{p}-z_{q}\right)}^{2}/h^{2}}e^{-{||N(x_{p}^{t})-N(x_{q}^{t})||}^{2}/kh^{2}},$$

where *z* is the HR reference data, *p* and *q* are data indexes, *x* ^*t*^ is the current reconstructed data at iteration *t (being x* ^0^ the initial interpolated version of LR data), Ω is a 3D search area, *N*(*x*_*i*_^*t*^) is a 3 × 3 × 3 voxel window surrounding reconstructed voxel *i* at iteration *t*, *h* and *k* parameters control the filtering strength and *C*_*p*_ is the normalization factor. 

The weights used during the reconstruction process are based on a combination between a sigma filter in the denoised HR image (first term in (6)) and a nonlocal means filter in the denoised and interpolated LR image (second term in (6)). Higher weights are thus given to voxels with similar intensity in the HR image and with similar local context in LR image at the same time. This strategy enables to take advantage of the information redundancy present in LR image as well as to use the structural information of the HR image to drive the reconstruction process. When a structure is only present in one of the images or has different signal properties (e.g., multiple sclerosis lesion), the reconstruction process is only driven by the image containing the most suitable information since the weights derived from the other image are nondiscriminating (i.e., all voxels have similar weights in homogeneous areas). By this way, the proposed method is robust to reconstruction artifacts such as ghosting structures. This aspect will be further discussed in experiment part where experiments on phantom with MS lesion are proposed.

(2)* Mean Correction.* In order to take into account the MR acquisition properties, we impose the constraint that the downsampled version of the reconstructed LR data must be equal to the original LR data. To ensure that, the mean value of the reconstructed HR voxels needs to be corrected to fit the value of the original LR voxel. This is accomplished by adding the corresponding offset to each reconstructed voxel:

(7) $${\hat{x}}^{t+1}={\hat{x}}^{t+1}-\text{NN}\left(DH{\hat{x}}^{t+1}-y\right),$$

where NN is the Nearest neighbor interpolation operation.

**Figure 1:**
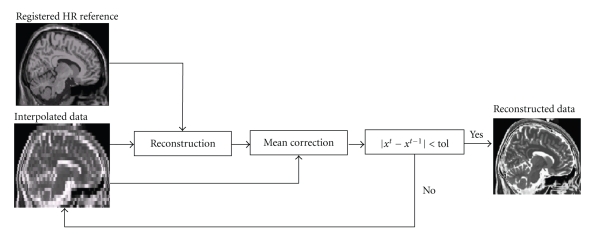
Block diagram of the proposed method. The registered HR reference data is used reconstruct the LR data iteratively until no significant difference is found between two consecutive reconstructions.

These two steps are iteratively repeated (decreasing the strength of the filtering each time) using the current reconstructed data in the next reconstruction step (instead of the initial interpolated data) until no significant difference is found between two consecutive iterations (mean absolute difference between two iterations is inferior to a given tolerance, *tol*). A block diagram of the proposed method can be observed in Figure 1.

## 3. Experiments and Results

### 3.1. Experimental Data

To validate the proposed method, a synthetic dataset was used. High resolution T1-w and T2-w datasets with both normal and pathology (multiple sclerosis) from the publicly available Brainweb database were used [15, 16]. The HR T1-w and T2-w volumes have 181 × 217 × 180 voxels with a resolution of 1 mm^3^. The Peak Signal-to-Noise Ratio (PSNR) measure was used to compare the reconstructed data and the original HR data (note that PSNR is equivalent to RMSE as it is an RMSE-derived measure but range invariant which is useful for comparing the results of images with different quantization levels). All the experiments were performed using Matlab 7.4 (Mathworks Inc.). Example data and source code of the proposed method can be downloaded from http:/personales.upv.es/jmanjon/reconstruction/super.html in order to enable the reproducibility of this work.

### 3.2. Implementation Details

As the proposed methodology can be implemented in a number of different manners, we will discuss here how these different alternatives were selected prior the comparison of the proposed methodology with other methods.

#### 3.2.1. Initial Interpolation

**Figure 2:**
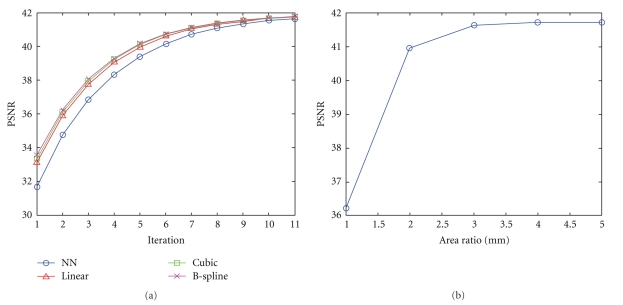
(a) Effect of initial interpolation method in the proposed method (all methods compared reached a similar stable result after 11 iterations). (b) PSNR values of the proposed method as a function of the radius of the search area. As can be noted, no significant improvement is found beyond that using a radius equal to 3.

To find out how the initial interpolation affects the reconstruction results of the proposed method, different interpolation methods (Nearest Neighbor, Trilinear, Cubic, and B-spline interpolation) were compared for the initial step. Results are shown in Figure 2(a). As can be noted, the proposed method obtained nearly the same stable solution in all the cases, independently of the initial interpolation method used in approximately the same number of iterations. Thus, a Nearest Neighbor interpolation seems to be the better option since it has the simplest and fastest implementation.

#### 3.2.2. Size of Search Volume

The proposed methodology was applied with different search area sizes and the results were analyzed (see Figure 2(b)). From this experiment, we found that a search volume Ω of size 7 × 7 × 7 voxels (3D window radius = 3) was the best option. Increasing the search volume beyond this size provides only a slight improvement while considerably increasing the computation time.

#### 3.2.3. Value of *h* and *k* Parameters

The value of *h* parameter plays a major role in the reconstruction process; thus its correct adjustment is very important. In the present method, an iterative decremental assignment of its value is proposed. This approach enables a stable *coarse to fine* reconstruction in a similar manner as done for Nonlocal demosaicing [17]. In this approach, the use of high values of *h* produces the averaging of different parts of the image while small values directly copy similar values. For 8-bit quantization input data, decreasing values of *h* (32, 16, 8, 4, and 2) were used in all experiments. Each value is used once and then decreased until the last *h* value (2 in our case) and then the process is iterated with *h* = 2 until the mean absolute value of the difference between two consecutive reconstructions falls below a given tolerance. For other quantization levels *h* values can be linearly adjusted. 

Regarding the *k* parameter, if this parameter is too small, the method will be very robust to misregistration but few improvements in the reconstruction will be achieved since almost not information from HR data will be used. In contrast, if *k* is too high, only HR information will be used which will lead in a good reconstruction when data is perfectly registered but the robustness of the method can be seriously affected when LR and HR data is geometrically incoherent. We have found experimentally that a factor *k* = 256 enables to obtain good reconstructions while maintaining the robustness of the method.

#### 3.2.4. Computational Complexity

Since the proposed approach is an iterative process, the computational burden of the method is high (being the filtering step the heaviest part). To reduce the processing time, we have implemented the proposed method using symmetric weight computation on the filtering step which reduces the computational burden a factor 2. Besides, a multithreading implementation was used which allowed to reduce the processing time another factor 4 in the Quad Core 2.4 GHz Pentium machine used in the experiments. This lets an average time of around 8 minutes per iteration. This makes the required time for reconstructing a typical MR volume to be approximately 1 hour. Further time reduction can be achieved by processing only object voxels avoiding useless computations at background voxels. 

To summarize, in all the experiments the search volume Ω in the HR volume was set to have a radius equal to 3 (i.e., a 3D region of 7 × 7 × 7 voxels); the size of the local 3D neighborhoods used to compute the similarity in the reconstructed LR images was set to 3 × 3 × 3 voxels and *h* = [32, 16, 8, 4, 2]. The parameter *k* was set to 256 and the *tolerance* was set to 0.01 (0.005% of the image range).

### 3.3. Comparison on Normal Brain Anatomy

**Figure 3:**
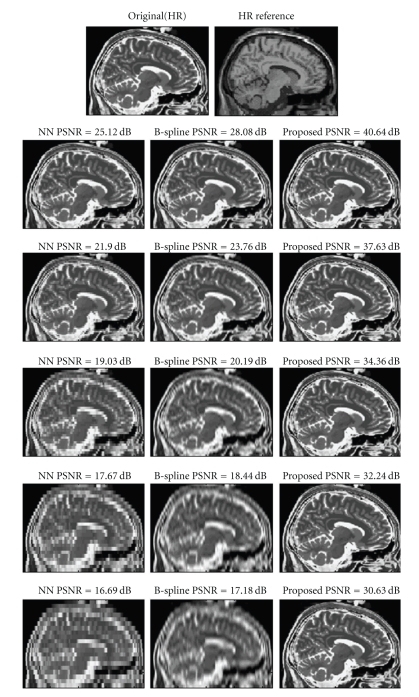
A sagittal slice for the normal anatomy case. From top to bottom, results with different slice thicknesses (2, 3, 5, 7, 9 mm) and from left to right, the NN reconstruction, the B-Spline reconstruction, and the proposed method.

The first comparison consisted in reconstructing Brainweb HR T2-w volume from their downsampled versions using HR T1-w data as HR reference. The HR T2-w volume was downsampled in the *z* direction to have different slice thickness (2, 3, 5, 7, and 9 mm). The Brainweb HR T1-w data used as reference had 1 mm^3^ voxel resolution. The resulting reconstructed data was compared to the Nearest Neighbor (NN) and B-Spline interpolation as implemented on MATLAB 7.4. In addition, the authors of the method recently proposed in Rousseau [8] provided results for the 3 mm slice thickness case for comparison. In these experiments, no noise was added to simplify the analysis of the results. It has to be noted that in general the proposed method is always applied after a denoising step; thus zero noise condition can be nearly met. The results can be observed in Table 1 and Figure 3. As can be noticed the proposed method drastically improved the results in all the cases.

### 3.4. Comparison on Pathological Brain Anatomy (Multiple Sclerosis)

**Figure 4:**
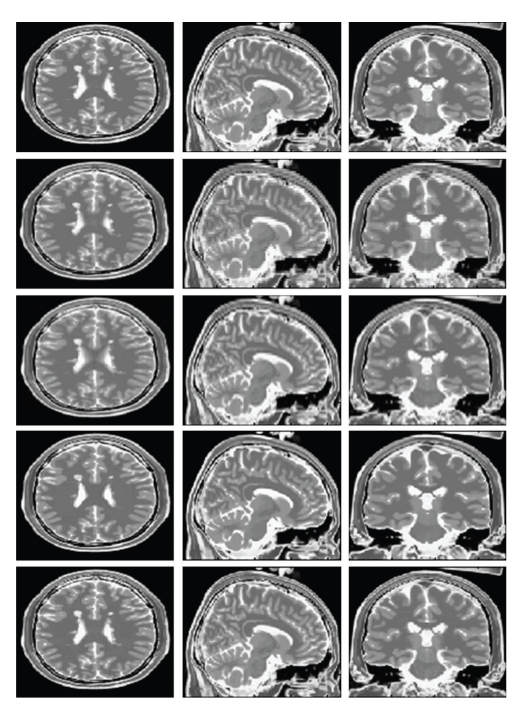
From top to bottom: Original Axial, Sagittal and Coronal sections of the HR T2 data, NN interpolation, B-Spline interpolation, Rousseau's method and proposed reconstruction. We have the results for 3 mm slice thickness reconstruction. Note that MS lesions are better reconstructed using the proposed method.

In this case, the same experiment was repeated as above, but this time using the MS T2-w HR and MS T1-w HR phantoms available from the Brainweb website. The proposed method was also compared to the NN and B-Spline interpolation. Again, Rousseau's method results for 3 mm slice thickness were supplied by the authors. The results can be observed in Table 2. In Figure 4, a visual comparison of the results for 3 mm slice thickness can be done. Once more, the proposed method drastically improved the results in all the cases. 

We were curious about the effect of the proposed method in the MS lesions, since lesions appearing as T2-w hyper intensities are not often clearly visible in T1-w. We observed that MS lesions were well reconstructed, even though the T1-w regularization information did not help much to recover such structures. This fact can be understood taking into consideration the fact that the proposed method extracts information not only from the HR reference data but also from redundant patches in the LR data.

### 3.5. Noise Sensitivity

It is clear that the zero noise case is an idealization of the real MR image conditions. To address this issue, another experiment was performed on the Brainweb data, but this time adding Rician noise. An LR T2-w volume of voxel resolution 1 × 1 × 5 mm was reconstructed to 1 mm^3^. Both HR reference T1-w and LR T2-w data were corrupted with several levels of Rician distributed noise (0, 1, 2, and 4% of the maximum intensity). The noiseless phantoms were considered to be complex valued with the imaginary part equal to zero for the generation of Rician noise. Noise was generated by adding Gaussian noise to the real and imaginary parts and then computing the complex modulus, thus forming a magnitude image.

**Figure 5:**
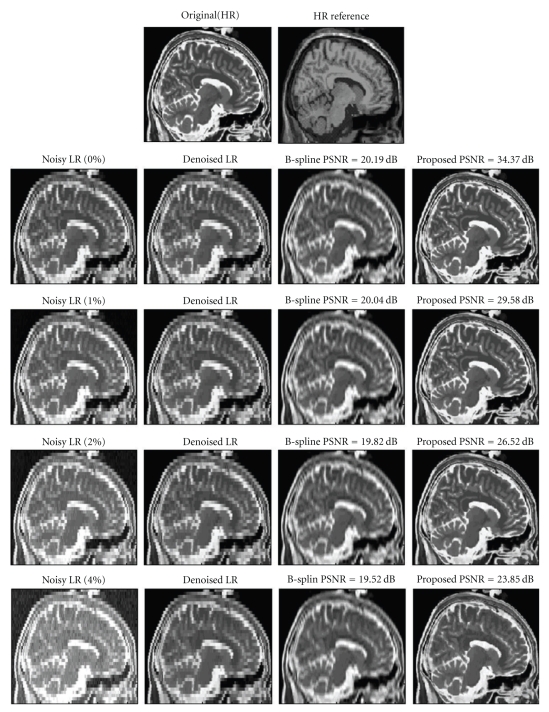
From Top to Bottom, 0, 1, 2, and 4% noise case. From left to right, LR noisy T2-w data, LR-denoised T2-w data, B-spline interpolation, and proposed reconstruction.

To ensure a fair comparison, NN and B-Spline interpolation methods used also the denoised data to compute the HR T2-w volume. Qualitative results are shown in Figure 5 and quantitative results are presented in Table 3. 

Again, the proposed method outperformed the other methods in all noise levels. One can notice that performance of the proposed method decreases with the noise amplitude as the denoising process inevitably erases some high-frequency information in the images. It is also important to note that in this experiment we have added the same level of noise to LR and HR data as it is supposed to be machine dependent, but in many cases HR data is acquired with 3D acquisitions and therefore can be less noisy than an LR multislice acquisition (e.g., a typical T1-w volume compared to DWI-MRI). In such cases, the reconstruction of the LR data is highly improved due to the fact that the HR data does maintain its high-frequency information.

### 3.6. Registration Sensitivity

As it has been pointed out previously, the accuracy of the proposed method highly depends on the correct registration of the LR and HR images. To evaluate how the misalignment affects the accuracy of the proposed method, the HR T1-w reference volume was shifted in the 3 directions (*x*, *y*, and *z*). In this experiment, a 1 × 1 × 5 mm voxel resolution LR T2-w volume was reconstructed to 1 mm^3^ voxel resolution using as reference a randomly shifted HR T1-w volume. The experiment was performed by applying random shifts in all 3 directions by a certain amount (1, 2, and 3 mm) and the experiment was repeated 10 times for each shift value to estimate the variability of the method. Results are shown in Table 4. In this case the IPSNR measure was used which represents the improvement of the PSNR of the proposed method compared to the B-Spline method which was taken as reference.

From these results, it can be concluded that the proposed method is able to tolerate a small misregistration (up to 1 or 2 mm) while maintaining an improved performance over the reference B-Spline interpolation. This is mainly due to the inclusion of information of LR data during reconstruction. This is important since in real world conditions small misalignments can be present after registration. However, most of current linear registration methods are able to obtain a sub-millimeter accuracy which enables the application of the proposed methodology [18].

### 3.7. Real Clinical Data

To evaluate quantitatively and qualitatively the proposed approach on real clinical data, three real datasets were used. In the three cases, the 3D search region was set to 7 × 7 × 7 voxels, the *h* values used were consecutively 12%, 6%, 3%, 1.5%, and 0.7% of the HR image range, and the tolerance was set to the 0.0005%. 

**Figure 6:**
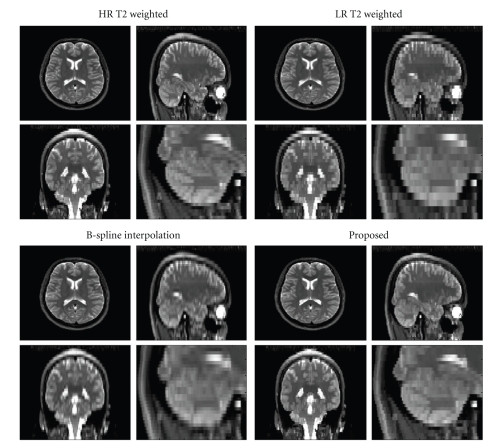
Comparison on real clinical data. Top-Left: HR T2-w volume. Top-Right: downsampled version of the HR T2-w volume. Bottom-Left: B-spline reconstruction. Bottom-right: reconstruction using the proposed method. Note that the proposed methodology yields a significantly less blurred reconstruction than other methods compared. A close-up of the cerebellum area clearly shows the improved reconstruction.

The first case consisted in a dual PD-w/T2-w study. In this way, we were sure that PD-w and T2-w data were perfectly registered as they are acquired at the same time. This dataset was obtained with a PD-w/T2-w volumetric sequence (256 × 256 × 56 voxels with a voxels resolution of 0.94 × 0.94 × 3 mm) in a Philips Gyroscan 1.5 Tesla scanner (The Netherlands). In this case, the T2-weighted volume was downsampled to a voxel resolution of 0.94 × 0.94 × 6 mm (i.e., a reduction factor 2 in *z* direction). Both reference PD-w and T2-w volumes were filtered using the BNLM3D method and PSNR values were computed using as reference the denoised version of the HR T2-w volume. The NN interpolation method obtained a PSNR equal to 27.68 dB, the B-Spline method 28.90 dB, and the proposed method 32.51 dB. In Figure 6, the different results can be visually compared. One can see that the reconstruction using the proposed approach not only obtained a better PSNR value than the other methods but also showed a better anatomical content.

**Figure 7:**
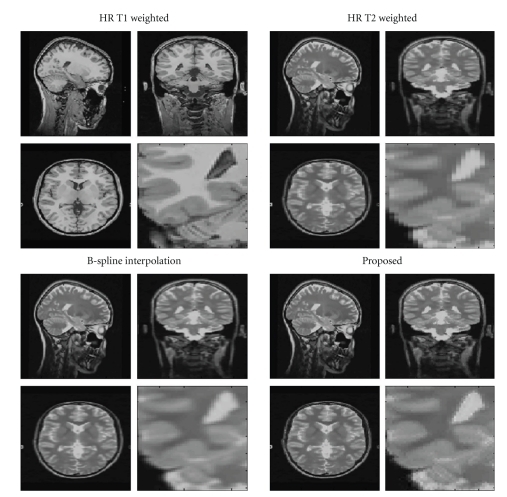
Comparison on real clinical data. Top-Left: HR T1-w volume. Top-Right: LR T2-w volume. Bottom-left: B-Spline reconstruction. Bottom-right: reconstruction using the proposed method. In each case a close-up is presented to better show how the proposed method provides a more consistent and less blurry reconstruction.

The second dataset consisted in an HR T1-w (170 × 256 × 256 voxels) and an LR T2-w (85 × 256 × 256 voxels) images acquired on a 3T Siemens Tim Trio (Erlangen, Germany) machine. The resolution of the T1-w data was 1 mm^3^ while the resolution of the T2-w data was 2 × 1 × 1 mm^3^. In this case, the LR data was upsampled to 1 mm^3^ using the B-Spline and the proposed method.  Figure 7 shows the reconstruction results using the compared methods. As in this case we had not HR to compare, the results were judged visually. Such qualitative analysis showed that the reconstruction using the proposed approach was less blurry than B-Spline interpolation. To apply the proposed method the LR and HR were first filtered using the BMNLM3D filter and then the LR T2-w data was registered to the HR T1-w data using SPM5 software [19] with a 3D rigid transformation. 

**Figure 8:**
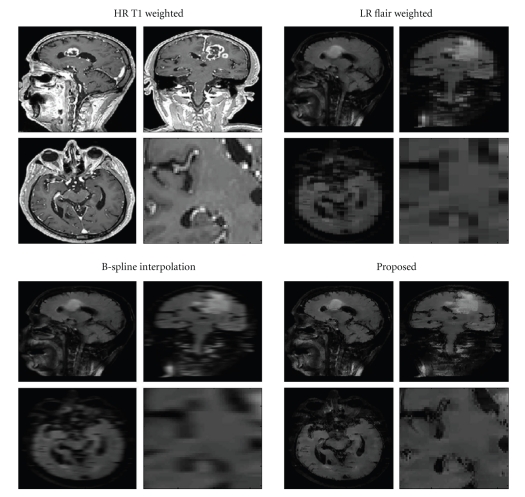
Comparison of real clinical data experiment. Top-Left: HR T1 data. Top-Right: LR FLAIR volume. Bottom-left: B-Spline reconstruction. Bottom-right: reconstruction using the proposed method. In each case a close-up of the coronal slice is presented to better show how the proposed method provides a less blurry reconstruction.

Finally, the third dataset consisted of a pathological dataset containing a brain tumor. In this case, an HR T1-w volume (224 × 256 × 174 voxels) and an LR FLAIR-w volume (224 × 256 × 29 voxels) were used. The resolution of the T1-w data was 1 mm^3^ while the resolution of the FLAIR data was 1 × 1 × 6 mm^3^. This dataset (named CEREBRIX) was downloaded from a public MR DICOM data repository (http://pubimage.hcuge.ch:8080/). Again, the LR data was upsampled to 1 mm^3^ using the B-Spline interpolation and the proposed method and the results were qualitatively evaluated. In Figure 8, the reconstruction results are compared to B-Spline reconstruction. Again, the visual inspection of the results showed a less blurry reconstruction when using our proposed approach showing consistent anatomical information. To apply the proposed method, the data was filtered using the BNLM3D filter and the LR FLAIR data was registered to the HR T1 data using also the SPM5 software with a 3D rigid transformation. 

## 4. Conclusion

We have presented a new superresolution method that enables the recovery of HR data information from LR data when coplanar HR data volume of the same subject from the same or other modality is available. The proposed method has been validated, using synthetic and real datasets. Our experiments demonstrated that the proposed method outperforms classical state-of-the-art interpolation methods.

We have presented several experiments where images with highly anisotropic voxels have been reconstructed to have isotropic voxels (e.g., 1 × 1 × 9 mm^3^ to 1 mm^3^) when HR data of such resolution is available. It is worth noting here that this reconstruction can be performed in any dimension when suitable data is available (e.g., 3 × 3 × 3 mm^3^ to 1 × 1 × 1 mm^3^). 

Our iterative approach relies on a correct registration of LR and HR data to assure that HR similarities can be extrapolated to help in the reconstruction of LR data. However, it was shown that the proposed method is robust to a small misregistration. Moreover, a proper denoising step is mandatory prior to the reconstruction process. In this sense, we have used a BNLM3D method that performed very well in all cases. 

The use of a 3D boxcar function as convolution matrix used in the mean correction step is supported by the concept of partial volume on MRI where voxel intensity can be modeled as a linear combination of the voxel intensities of the adjacent HR data. Our experiments on real data seem to confirm this assumption yielding plausible results when using this model. 

It is important to note that no special hardware or specific imaging sequences are needed to apply the proposed approach. The proposed methodology can be applied to increase the resolution of multimodal studies after data registration. This can potentially benefit multispectral MR data segmentation and the extraction of multimodal features from the reconstructed data. 

Other specially interesting application could be the application of the proposed technique to artificially increase the resolution of fMRI or DTI studies where typically an HR reference volume is acquired together with the LR functional data.

## Figures and Tables

**Table 1:** PSNR values (larger values are better) of the different methods compared for several slices thicknesses for the normal brain anatomy case.

Slice Thickness (mm)	2	3	5	7	9
NN	25.13	21.91	19.04	17.68	16.70
B-Spline	28.09	23.77	20.20	18.44	17.18
Rousseau	—	26.71	—	—	—
Proposed	**40.65**	**37.64**	**34.37**	**32.24**	**30.64**

**Table 2:** PSNR values of the different methods compared for several slices thicknesses for the multiple sclerosis anatomy case.

Slice Thickness (mm)	2	3	5	7	9
NN	26.21	22.99	20.09	18.72	17.73
B-Spline	29.33	24.99	21.29	19.59	18.33
Rousseau	—	27.33	—	—	—
Proposed	**41.04**	**38.08**	**34.81**	**32.64**	**30.96**

**Table 3:** PSNR values of the different methods compared for several noise levels.

Noise (%)	0	1	2	4
NN	19.04	18.93	18.78	18.56
B-Spline	20.19	20.03	19.82	19.52
Proposed	**34.37**	**29.58**	**26.52**	**23.85**

**Table 4:** Average IPSNR values of the proposed referred to B-Spline method in function of misregistration.

Random Shift (mm)	0	1	2	3
IPSNR	13.92	5.12 ± 0.25	0.53 ± 0.35	−0.09 ± 1.47
